# Local Dose Effects for Late Gastrointestinal Toxicity After Hypofractionated and Conventionally Fractionated Modern Radiotherapy for Prostate Cancer in the HYPRO Trial

**DOI:** 10.3389/fonc.2020.00469

**Published:** 2020-04-03

**Authors:** Wilma D. Heemsbergen, Luca Incrocci, Floris J. Pos, Ben J. M. Heijmen, Marnix G. Witte

**Affiliations:** ^1^Department of Radiation Oncology, Erasmus Medical Center, Rotterdam, Netherlands; ^2^Department of Radiation Oncology, The Netherlands Cancer Institute, Amsterdam, Netherlands

**Keywords:** prostate cancer, hypofractionation, gastrointestinal toxicity, dose-surface maps, radiotherapy, NTCP

## Abstract

**Purpose:** Late gastrointestinal (GI) toxicity after radiotherapy for prostate cancer may have significant impact on the cancer survivor's quality of life. To date, little is known about local dose-effects after modern radiotherapy including hypofractionation. In the current study we related the local spatial distribution of radiation dose in the rectum to late patient-reported gastrointestinal (GI) toxicities for conventionally fractionated (CF) and hypofractionated (HF) modern radiotherapy in the randomized HYPRO trial.

**Material and Methods:** Patients treated to 78 Gy in 2 Gy fractions (*n* = 298) or 64.6 Gy in 3.4 Gy fractions (*n* = 295) with available late toxicity questionnaires (*n* ≥ 2 within 1–5 years post-treatment) and available 3D planning data were eligible for this study. The majority received intensity modulated radiotherapy (IMRT). We calculated two types of dose surface maps: (1) the total delineated rectum with its central axis scaled to unity, and (2) the delineated rectum with a length of 7 cm along its central axis aligned on the prostate's half-height point (prostate-half). For each patient-reported GI symptom, dose difference maps were constructed by subtracting average co-registered EQD2 (equivalent dose in 2 Gy) dose maps of patients with and without the symptom of interest, separately for HF and CF. *P*-values were derived from permutation tests. We evaluated patient-reported moderate to severe GI symptoms.

**Results:** Observed incidences of rectal bleeding and increased stool frequency were significantly higher in the HF group. For rectal bleeding (*p* = 0.016), mucus discharge (*p* = 0.015), and fecal incontinence (*p* = 0.001), significant local dose-effects were observed in HF patients but not in CF patients. For rectal pain, similar local dose-effects (*p* < 0.05) were observed in both groups. No significant local dose-effects were observed for increased stool frequency. Total rectum mapping vs. prostate-half mapping showed similar results.

**Conclusion:** We demonstrated significant local dose-effect relationships for patient-reported late GI toxicity in patients treated with modern RT. HF patients were at higher risk for increased stool frequency and rectal bleeding, and showed the most pronounced local dose-effects in intermediate-high dose regions. These findings suggest that improvement of current treatment optimization protocols could lead to clinical benefit, in particular for HF treatment.

## Introduction

Irradiation of tumors in the pelvic area through external beam radiotherapy comes inevitably with dose delivery to nearby organs at risk, such as the rectum. The potential permanent impact of late gastrointestinal (GI) toxicity after radiotherapy may have significant impact on the cancer survivor's quality of life (1). Preventing chronic late GI toxicity is therefore critical. For this purpose understanding how we should distribute radiation dose to surrounding normal tissues while keeping toxicity risks as low as possible is critical.

The QUANTEC project (quantitative analysis of normal tissue effects in the clinic) previously summarized the available clinical data and models on acute and late radiation-induced toxicities with the goal to improve patient care by providing useful tools (2). These models were mainly derived from traditionally fractionated 3D conformal radiotherapy (3DCRT). Shortcomings and open issues of the available models have broadly been recognized, including the uncertainty of fractionation effects, a lack of reliable models for modern radiotherapy with IMRT dose distributions and image-guidance, and a lack of knowledge concerning spatial effects (3). This causes a number of deficits in current strategies of treatment planning optimization in the current era of IMRT, image-guidance, and hypofractionated treatment.

With respect to the inhomogeneous dose distributions in the rectum, achieved with either radiation technique or fractionation schedule, we can theoretically translate physical dose distributions into (radio)biological dose parameters using mathematical models derived from radiobiology (4, 5). Altered fractionation schedules in recent hypofractionation trials in prostate cancer are based on such models (6–9). However, to achieve reliable biological NTCP models for late GI toxicity after modern RT, we first have to gain insight into local dose-effects and (hypo)fractionation effects in real patient populations rather than depending solely on theoretical models.

Historically, dose-response for normal tissues were evaluated taking dose-volume distributions to a whole single organ into consideration. It is nowadays recognized that function and radiosensitivity may vary within an organ, and that dose-shapes might be relevant. Therefore, local spatial dose evaluations beyond the boundaries of delineations and dose-volume may enhance our understanding of mechanisms causing radiation-induced damage (10). In particular voxel-based dose mapping procedures have been introduced to take into account the spatial dose distribution by co-registering dose distributions to a region of interest, often using a template patient. For hollow organs such as the rectum, a spatial 2D dose distribution of the rectal wall (i.e., virtual unfolding of the rectum to a 2D structure) is considered reasonably sufficient for this purpose (11–18). Evaluation of local rectal and anal dose distributions in relation to acute and late gastrointestinal toxicity endpoints by means of dose mapping have been previously performed by several research groups. This concerned mainly patients treated with conventional fractionation schedules, identifying local dose effects for various endpoints including rectal bleeding, fecal leakage, and increased stool frequency (11–18).

In the current study we explored local rectal dose distributions and their relation to GI toxicity endpoints, for both hypofractionated (HF) and conventionally fractionated (CF) treatment, using toxicity data and planning data from the HYPRO trial. In this trial patients were randomized between conventional and hypofractionated treatment, delivered with modern radiotherapy techniques including IMRT, image-guidance, and online prostate position verification.

## Materials and Methods

### Patient Selection

The dataset of a recent Dutch randomized clinical trial (HYPRO) was analyzed in which patients were randomized to 78 Gy in conventional 2 Gy fractions (CF) or 64.4 Gy in hypofractionated 3.4 Gy fractions (HF) (19). Selected patients were eligible for the current study in case both late toxicity data (*n* ≥ 2 questionnaires within the period 1–5 year post-treatment (*N* = 633, Table 2) and 3D planning data were available (which were not available for 40 patients), leaving 593 patients for the current study. Because planning of patient visits may vary from the study schedule, we accepted questionnaires up to 5.5 year post-treatment.

### Treatment

Based on an estimated α/β for prostate cancer of 1.5 Gy, the EQD2 was 90.4 Gy for HF vs. 78.0 Gy for CF. For normal rectal tissue with an estimated α/β of 3 Gy, the EQD2 was 82.7 Gy for HF vs. 78.0 for CF. The clinical target volume was the prostate with or without the seminal vesicles (SV): based on the estimated risk of SV involvement according Partin tables (20), a SV dose of 0 Gy, 72.2 Gy, or 78 Gy was planned (19). The outer contours of the rectum were delineated on the planning CT scan from the anal verge to the bottom of the sacro-iliac joints. The HYPRO protocol prescribed that the rectal volume receiving 83% of the prescribed dose should be below 50% for the total rectal volume or below 60% for the rectal wall. Further treatment optimization was performed in accordance with local protocols at each participating center. The applied treatment technique for 99% of the patients was image-guided IMRT with daily online positioning of the prostate. For this purpose, cone beam CT was used in 23% and portal imaging devices was used in 77% of the cases. A small proportion was treated with 3DCRT (1%). One center applied a rectal balloon, which pushes the posterior rectal wall out of the intermediate dose region (21). Further details of treatment planning have been previously reported (6, 19). CF patients received 5 fractions per week, and HF patients 3 fractions per week with 1 day intervals (Monday, Wednesday, Friday).

### Toxicity Endpoints

The patient-reported GI symptoms were extracted from a patient-reported symptom list (questionnaire) distributed in the HYPRO trial at the late time points of 6 months, and yearly between 1 and 5 year (22). Evaluated GI symptoms were: rectal bleeding, fecal incontinence, pain/cramps with stools, mucus discharge (all had to be reported as moderate or severe to be scored), and increased stool frequency ≥ 4 per day. We identified from all available questionnaires the maximum score for each toxicity endpoint of interest.

### Dose-Surface Maps

For the rectal wall the dose surface mapping was based on a central axis which was first computed as the maximum of a Euclidean distance transform. The average length of the delineated rectum along the central axis was 14.9 cm for both HF and CF. The intersections of equidistant slices perpendicular to this axis with the delineated rectum surfaces provided the corresponding locations between patients. We calculated two types of dose surface maps: (1) “total rectum mapping”: the delineated rectum with its central axis scaled to unity, and (2) “prostate half mapping”: the delineated rectum next to the prostate with a length of 7 cm along the central axis (plus 4 cm in cranial direction and minus 3 cm in caudal direction, measured from the half-height position of the prostate). These cutoffs were chosen to cover the dose range in the rectum of about 50–100% of the prescribed dose.

To correct a patient averaged dose-surface map for fractionation effects using the linear-quadratic model (i.e., equivalent dose in 2 Gy: EQD2), we applied a chosen α/β ratio of 3 Gy to the dose distribution of each patient. The resolution of the dose maps was chosen to effectively slightly exceed a 2 mm dose grid resolution. In the circumferential direction 90 pixels were taken, i.e., every 4 degrees. In the axis direction of rectum maps 100 pixels were taken, which would effectively cover a 15 cm long rectum at 1.5 mm resolution. As a final step, resulting dose-surface maps of individual patients (physical and biological) could be averaged and subtracted for each identified toxicity endpoint (yes vs. no). Further details have been previously reported (14).

### Statistical Analysis

Distributions of baseline characteristics within the HF and CF group were calculated and tested for differences applying a Chisquare test for the ordinal and binary variables, and a *T*-test for age. Associations between clinical covariates and toxicity endpoints were tested univariate using binary logistic regression. For each evaluated GI symptom, dose difference maps were constructed by subtracting average EQD2 dose maps of patients with and without the toxicity of interest, separately for HF and CF. For the calculation of a *p*-value for each dose difference map, we used a permutation approach, randomly re-shuffling the patients among the subgroups (23). For the determination of significant differences within a dose-difference map, we calculated and evaluated the false discovery rates “q” as a realistic estimate of the local *p*-values, which is a practical and powerful approach to tackle the multiple testing issue (24, 25).

## Results

### Baseline Characteristics

The baseline characteristics of the selected study patients are summarized in Table 1 which shows that distribution of the characteristics are similar for HF and CF except for a history of TURp which was more common in the CF group (11 vs. 7%, *p* = 0.07, Table 1). A history of TURp was however not associated with any of the evaluated moderate to severe GI symptoms.

**Table 1:** Patient and treatment characteristics (*N* = 593).

**Variable**	**CF (*n* = 298)**	**HF (*n* = 295)**	***p-*value^**#**^**
Age (mean, sd) in years	70.1 (6.0)	69.5 (6.6)	0.2
TURp	11%	7%	0.07
Abdominal surgery	26%	25%	0.7
Diabetes mellitus	13%	14%	0.5
Adjuvant hormonal therapy	65%	63%	0.6
Fiducial markers	95%	95%	0.9
IMRT	98%	99%	0.3
**T category**			
T1-2	46%	48%	0.7
T3-4	54%	52%	
**PTV margins prostate**			
5-7 mm	89%	89%	0.9
8-10 mm	11%	11%	
**Dose seminal vesicles**			
0 Gy	23%	20%	0.4
72-78 Gy	77%	80%	

# *p-values calculated with Chisquare test, except for age (t-test)*.

### Reported GI Toxicities

In Table 2, the observed incidences of the late GI toxicities of interest are summarized per treatment group, both for all patients in the HYPRO trial who filled out ≥2 late questionnaires (*N* = 633) and for the selected group with available CT scans and dose distributions (*N* = 593). These are the result of accumulation over all available questionnaires between Year 1 and Year 5, taking maximum scores. The table shows that the selected population with available dose information, was a non-biased and representative selection of the patient group that filled out late questionnaires.

**Table 2:** Incidence of late gastrointestinal toxicity endpoint (evaluated by the patient as “moderate—severe”) on questionnaires in the period 1–5 year post-treatment.

	**≥2 questionnaires (*****N*** **=** **633)**			**With available dose maps (*****N*** **=** **593)**		
	**CF**	**HF**	**p**	**CF**	**HF**	**p**
	***n*** **=** **310**	***n*** **=** **323**		**n** **=** **298**	**n** **=** **295**	
**Late GI endpoint**						
Stool frequency ≥4/day	12.3%	19.5%	**0.013**	12.1%	19.7%	**0.011**
Rectal bleeding	11.0%	16.7%	**0.037**	10.7%	17.6%	**0.016**
Mucus discharge	5.2%	6.2%	0.6	5.0%	6.4%	0.5
Pain/cramps with stools	7.4%	9.9%	0.3	7.7%	10.2%	0.3
Fecal incontinence	10.6%	11.1%	0.8	10.7%	11.5%	0.8
≥1 symptom	30.3%	35.6%	0.16	30.2%	36.3%	0.12
≥2 symptoms	12.6%	18.0%	0.061	12.4%	19.3%	**0.020**
≥3 symptoms	3.2%	6.8%	**0.040**	3.4%	7.1%	**0.040**

*P < 0.05 are indicated in bold. GI, gastrointestinal; HF, hypofractionation; CF, conventional fractionation*.

About 35% of CF and HF patients experienced one or more moderate to severe late symptoms after modern RT, accumulated over the evaluated late period (Table 2). Compared to CF, significantly higher incidences after HF treatment were observed for the late endpoints of rectal bleeding and increased stool frequency. More HF patients experienced multiple moderate to severe GI symptoms.

Among the late GI endpoints of study, all endpoints showed significant correlations with the other ones (i.e., if a patient reported 1 symptom it was likely that he also reports one or more of the other symptoms). Highest correlations were observed between fecal incontinence-increased stool frequency, and rectal bleeding-mucus discharge (*p* < 0.001).

### Associations Between Clinical Covariates and Toxicity Endpoints

The results of assessing the associations between baseline covariates and the toxicity endpoints of interest are summarized in Table 3. Rectal incontinence was significantly associated with diabetes and age. Rectal bleeding and mucus discharge were significantly associated with T stage.

**Table 3:** Association between clinical baseline covariates and toxicity endpoints.

	**Stools** **≥** **4/day**		**Rectal bleeding**		**Mucus discharge**		**Pain/cramps**		**Fecal incontinence**	
	**HR**	***p***	**HR**	***p***	**HR**	***p***	**HR**	***p***	**HR**	***p***
Age > 70 vs. ≤ 70	0.87	0.5	0.89	0.6	0.61	0.2	0.63	0.1	2.67	**<0.01**
TURp yes vs. no	1.19	0.6	0.90	0.8	1.34	0.6	0.37	0.2	1.96	0.07
Previous abdominal surgery yes vs. no	0.76	0.3	1.23	0.4	0.47	0.1	1.14	0.7	1.26	0.4
Diabetes yes vs. no	1.21	0.05	0.84	0.6	no result		0.97	0.9	**2.05**	**0.024**
AHT yes vs. no	1.02	0.9	1.11	0.7	1.98	0.09	0.63	0.1	1.16	0.6
T3-4 vs. T1-2	1.00	1.0	**1.60**	**0.046**	**2.13**	**0.04**	1.00	1.0	0.87	0.6

*P < 0.05 are indicated in bold. Results from univariate logistic regression (N = 633). OR, Odds ratio; TURp, transurectal resection of prostate; AHT, adjuvant hormonal therapy; SV, seminal vesicles*.

### Dose-Surface Maps

**Figure 1:**
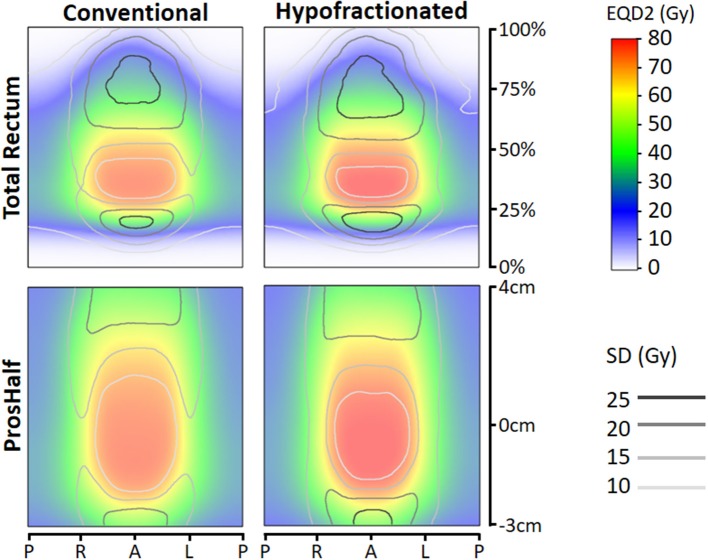
Total rectum (upper panes) and prostate-half (lower panes) mean dose-surface maps with distance along central axis (vertical) against location along circumference axis (horizontal). Left panes represent mean dose-surface maps of conventionally fractionated patients, right panes for hypofractionated patients. EQD2 = equivalent dose for 2 Gy fractions with α/β=3 Gy. *Abbreviations:* P, posterior; R, right; L, left; A, anterior; SD, standard deviation.

Figure 1 shows the average EQD2 dose-surface maps and local standard deviations for both types of mapping and for both groups (CF and HF). Comparing the EQD2 dose distributions of CF and HF, we observed that the high-dose area is darker red for HF which can be explained by the somewhat higher prescription dose in EQD2 for HF (82.7 Gy vs. 78 Gy). Furthermore, the rectal surface receiving dose levels in the range of ≥ 1–≥ 65 Gy EQD2 look very similar for HF and CF, whereas the rectal surface receiving dose levels in the range of > 65–≥ 80 Gy EQD2 were on average different with larger surfaces for HF. From previous calculations of “traditional” whole organ dose-surface histograms (DSH), it is known that indeed the average DSH of HF vs. CF only show a slightly unfavorable dose level in the range of > 65–≥ 80 Gy EQD2 (supporting DSH figure in Supplementary File). Furthermore, local standard deviations were larger for HF. The rectum adjacent to the prostate, as shown on the prostate-half maps, received dose levels in the range of 20–80 Gy, with the largest standard deviations (i.e., variation between patients) at the cranial and caudal side. The total rectum maps show dose levels in the range of 0–80 Gy, with 0–10 Gy in the most caudal 15% (the anal canal region) and the most cranial part close to the rectosigmoid region.

### Dose-Difference Maps

**Figure 2:**
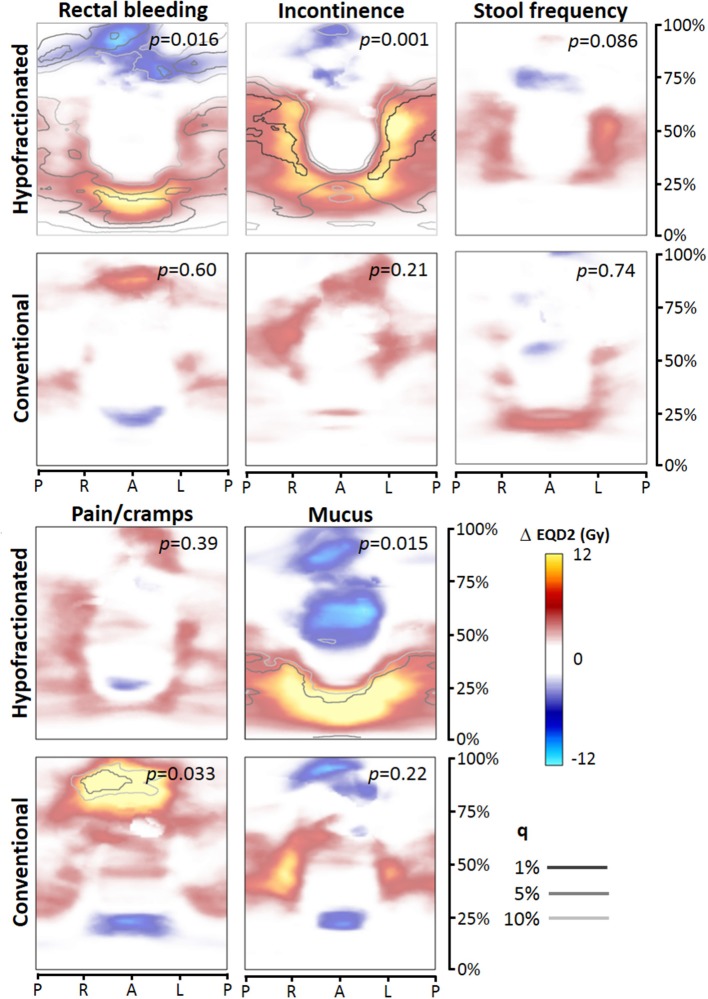
Dose difference maps (ΔEQD2) based on total rectum dose mapping, for the toxicity endpoints (moderate to severe), for the hypofractionated and conventional group separately. EQD2 = equivalent dose for 2 Gy fractions with α/β = 3 Gy, q = false discovery rate. *Abbreviations:* P, posterior; R, right; L, left; A, anterior.

**Figure 3:**
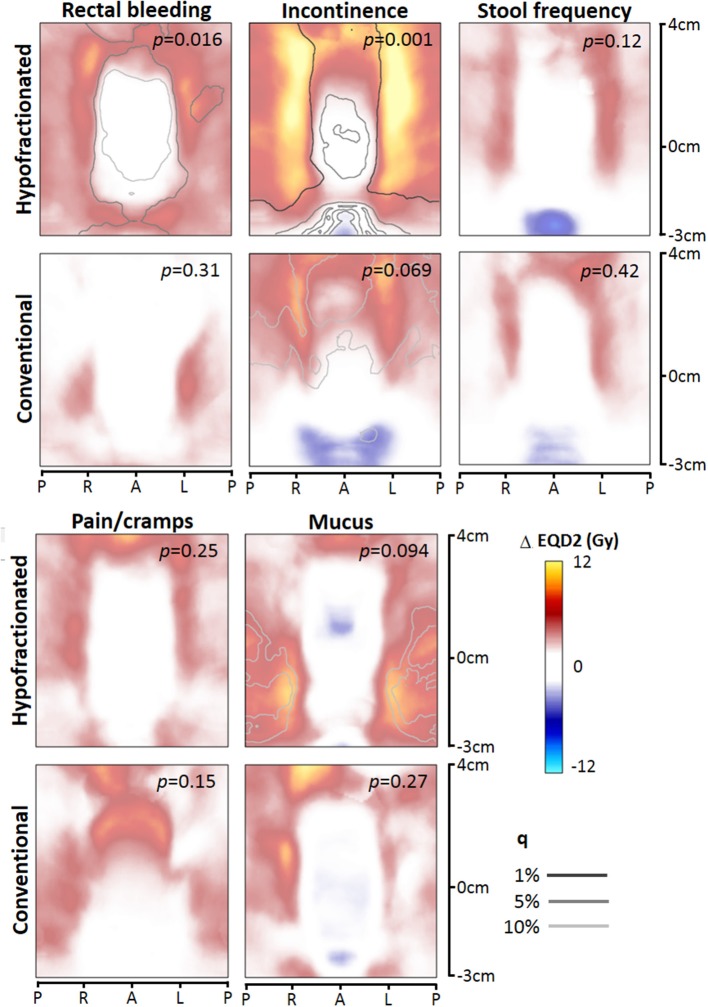
Dose difference maps (ΔEQD2) based on prostate-half height dose mapping, for the toxicity endpoints (moderate to severe), for the hypofractionated and conventional group separately. EQD2 = equivalent dose for 2 Gy fractions with α/β = 3 Gy, q = false discovery rate. *Abbreviations:* P, posterior; R, right; L, left; A, anterior.

For each toxicity endpoint, four dose difference maps were constructed: total rectum mapping and prostate-half mapping, and for each type of mapping the HF and CF version (Figures 2, 3). In general, one or more significant dose difference maps were obtained for all GI endpoints except for increased stool frequency (lowest observed *p* = 0.086). All dose-difference maps were also generated with physical dose instead of EQD2 dose, to check whether this might change results. Since they were very similar to the EQD2 versions, we report here only results based on EQD2 dose maps.

For rectal bleeding, large local dose differences (*p* = 0.016) up to ≥10 Gy were observed between patients with and without this complaint (Figures 2, 3), but only for HF patients. Remarkably, the prostate-half mapping (Figure 3) indicates significant differences in the region next to the prostate, whereas the total rectum mapping (Figure 3) indicates local dose-effects at a more cranial part of the rectum. Both locations are regions were on average ≈60 Gy (EQD2) is received by the rectal tissue (Figure 1).

For the late endpoint fecal incontinence, highly significant local dose-effects were found for the region receiving intermediate to high dose, i.e., in the neighborhood of the prostate (Figures 2, 3), but again only for HF patients. For mucus discharge, we also observed local dose-effects for HF patients only, which were identified by the total rectum mapping (Figure 2). Pain/cramps with stools was associated with local dose distributions in CF patients; for HF patients no such effect was observed (Figure 2).

## Discussion

We explored local dose-effect relationships for GI toxicity in a study population treated with both conventionally fractionated and hypofractionated modern radiotherapy. Since both patient groups were treated within the same randomized trial, this is a unique dataset to study hypofractionation effects on rectal toxicity with a perfect internal reference group of CF patients. We observed significant local dose-effect relations for all studied GI endpoints, except for increased stool frequency. For the endpoints rectal bleeding, pain/cramps, and mucus discharge, we observed differences between HF and CF in the patterns and level of significance of local dose-effects, whereas for pain or cramps with stools, observed patterns and levels of significance were similar.

We evaluated two types of dose mapping. The “total rectum” mapping is more accurate in matching specific anatomical sub-locations within the rectum between different patients an also covers the most cranial and caudal part of the rectum, whereas the “prostate-half” mapping is more accurate in matching the intermediate-high dose areas behind the prostate from one patient to another. The identified local dose-effects for both types of mapping were similar with comparable *p*-values. Theoretically, we expected that the prostate-half mapping would be more accurate in identifying risks associated with high-dose regions close to the prostate and is therefore of added value to the total rectum mapping which covers the whole anorectal tract, which was demonstrated in a previous study (14). However, we could not confirm this in the current study.

In the current study we used patient-reported toxicity from a prospective setting, accumulating the incidence over available questionnaires between year 1 and 5. As a result, 30% (CF) and 36% (HF) reported ≥1 moderate to severe complaint within this period. Previously, we reported that at 36 months of follow-up, 36% (CF), and 38% (HF) had a clinically relevant deterioration on the gastrointestinal subscale of the Prostate Cancer 25 Quality of Life module (26), which is in fair agreement with the current findings based on the symptom questionnaire. As discussed in this previous paper (26), reported toxicity incidences and differences between CF and HF in the HYPRO trial are unfavorable compared to the CHHIP trial (7), which may have been caused by differences in target definition (for most HYPRO patients inclusion of the seminal vesicles), different patient population (HYPRO patients were mainly high-risk patients), and especially by a greater difference in EQD2 dose levels (with an α/β of 3 for normal tissue): 78 Gy (CF) vs. 82.7 Gy (HF) for the HYPRO trial, and 74 Gy (CF) vs. 72 Gy (HF – 20 × 3) for the CHHIP trial.

As reported by several previous studies, prospective registration translates in general into relatively high incidences of toxicity when compared to studies where only physician-reported toxicities are used, as we also previously demonstrated for the HYPRO trial (19). When we compare our patient-reported rates of rectal bleeding and fecal incontinence with the recent study of Onjukka et al. who also used patient-reported late toxicity in a modern IMRT setting with mainly conventional fractionation and partly mild hypofractionation, the reported rates are very similar for both endpoints: about 10% in both studies (18).

We found that patient-reported GI toxicity incidences were higher for HF compared to CF with respect to the endpoints ≥3 symptoms, stool frequency, and rectal bleeding. Furthermore, we demonstrated that after converting both the HF and CF dose maps with the linear-quadratic model (with α/β of 3 Gy) to EQD2, we obtained very different dose-difference maps (Figures 2, 3) where we would expect similar local dose-effects. This suggests that just by calculating EQD2 for a HF schedule, this might not completely capture the biological effect of a HF treatment. There are several differences between the HF and CF group which might have contributed to the observations of both higher incidences and different dose-difference maps: **(a)** applied dose constraints were based on earlier studies with CF; **(b)** the rectum dose for HF was on average somewhat higher because of the higher EQD2 prescription dose of 82.7 Gy with α/β = 3; **(c)** local dose variations (standard deviations) were larger for the HF group; and **(d)** HF was delivered three times a week with 3.4 Gy fractions instead of 5 times a week 2 Gy fractions.

The symptom rectal bleeding was highly correlated with mucus discharge, which can be expected since both symptoms are the result of a radiation proctitis. In the literature, the endpoint of rectal bleeding has been extensively studied and modeled since it is regarded as a dose-limiting late toxicity (3). We observed a significantly higher incidence of patient-reported moderate to severe late rectal bleeding for HF compared to CF (17.6% vs. 10.7%). We previously reported the EORTC/RTOG grade ≥2 incidence of rectal bleeding (requiring clotting time), which was also higher for HF patients (5 vs. 2%, *p* = 0.11). (22). For rectal bleeding pronounced local dose-effects were observed in the dose-difference maps in the moderate to high-dose rectal regions close to the prostate, but only for HF patients. The location is in general in concordance with the literature based on conventional treatment, were high-dose regions above ≈60–70 Gy are found to be relevant for rectal bleeding. Applied dose constraints in the clinic are based on these published models (3). In the HYPRO trial, rectal volumes receiving ≥83% of the prescribed dose (i.e., ≥65 Gy for CF and ≥54 Gy for HF) had to be limited at treatment optimization to ≤ 50%. Our results suggest that for HF this planning criterion was suboptimal, resulting in increased risks of rectal bleeding. However, this observation might also be in part related to the higher EQD2 prescription dose of 82.7 Gy.

We observed similar fecal incontinence rates between CF and HF, but higher rates of increased stool frequency for HF (Table 2). At the same time, these complaints were highly correlated. In a recent study of Cicchetti et al. (27), comparing CF with mild HF (2.25–2.75 Gy per fraction), higher levels of fecal incontinence were observed for mild HF compared to CF. For the endpoints increased stool frequency and fecal incontinence, dose to other neighboring structures, such as pelvic floor muscles and nerves, might be relevant as well, as reported in several studies (28, 29). However, in other studies, similar rates of fecal incontinence were observed between 3DCRT and IMRT groups whereas the latter was associated with largely reduced dose levels to the anal canal region (27, 30) which is in the same region as the pelvic floor muscles.

As previously published, the results of the HYPRO trial were negative with respect to its hypothesis, i.e., non-inferiority with respect to Grade ≥2 toxicity and superiority with respect to freedom from failure could both not be demonstrated for the HF arm (6). Therefore, is this hypofractionation schedule of 19 times 3.4 Gy not recommendable or acceptable for clinical practice. However, for studying hypofractionation effects and dose-effect relationships these data are very useful. In current clinical practice, the hypofractionation schedule of the CCHIP trial (7) and the Widmark trial (9) have been adopted by centers worldwide, in which hypofractionated treatment is distributed over several weeks of treatment with intervals >24 h between fractions, similar to the HYPRO trial. To understand more about fractionation effects and effects of intervals between fractions on late (permanent) damage to normal tissues, additional modeling of the dose and outcome data from hypofractionation trials is essential. Recently, Wilkins et al. reported on dose-effect analyses from the CCHIP trial, derived from both conventional whole organ evaluation and from spatial dose mapping, aiming at formulating novel dose constraints for mild hypofractionation regimens in 3 Gy fractions (31). They report that different rectal dose constraints were obtained for different GI symptoms. In their study, spatial dose metrics did not improve prediction compared to dose-volume information.

Data from the hypofractionated trial arm of the HYPRO trial have been used for toxicity modeling using dose-volume data and additional features derived from texture analysis (32). They reported models for the GI symptoms of fecal incontinence and rectal modeling including clinical factors, dose-volume factors, and derived texture features. From other phase III randomized hypofractionation trials (6–9, 33), there are to our knowledge no publications yet on additional dose-effect modeling.

It is nowadays broadly recognized that incorporating spatial local dose information from voxel-based organ-at-risk calculations, in contrast to whole organ evaluations, has the potential to improve NTCP models and therefore improve the quality of derived planning constraints (10). Several studies have demonstrated that spatial local dose metrics are suitable for NTCP modeling of rectal toxicity compared to traditional dose-surface (DSH) and dose-volume histograms (DVH) (12–18). Recently, Casares et al. (34) reported on the superiority of spatial metric by comparing NTCP models; they concluded that predictability of patient-reported GI toxicity increased using spatial metrics compared to DSH/DVH metrics. The HYPRO data set is a very suitable dataset for bioeffect modeling of toxicity with the goal to obtain meaningful NTCP models and related dose constraints for optimized treatment planning with modern techniques including hypofractionation. An essential question to answer prior to the modeling is: how to summarize the inhomogeneous dose distributions into meaningful dose parameters for subsequent modeling. The dose maps resulting from this study clearly show that especially intermediate-high dose areas in the rectum are associated with a number of GI symptoms, especially for HF treatment. As previously described by Bentzen et al. (4), true equieffective dose levels (with the same bioeffect) result in identical toxicity risks. Further modeling of the HYPRO data, by constructing NTCP models based on calculated EQD2 dose for each group, may demonstrate whether the calculated EQD2 levels are equieffective or whether other biological factors have to be taken into account to calculate the true biological equieffective dose. Furthermore, relevant clinical covariates have to be incorporated into such models as well to improve the predictive power of such models. As shown in Table 3, for the endpoint fecal leakage (age and diabetes) and for the endpoints rectal bleeding and mucus (T stage) predictive clinical covariates were identified. Our ultimate goal is to use the current findings to develop a biological NTCP model that correctly incorporates fractionation effects, modeling the GI toxicity as a function of biological dose. This could then theoretically be applied to all types of dose distributions including different fractionation schedules.

In conclusion, we demonstrated significant local dose-effect relationships for patient-reported late GI toxicity in patients treated with modern RT. HF patients were at higher risk for increased stool frequency and rectal bleeding, and showed the most pronounced local dose-effects in intermediate-high dose regions. These findings suggest that improvement of current treatment optimization protocols could lead to clinical benefit, in particular for HF treatment.

## Data Availability Statement

The datasets used in this study are available on request. The corresponding author can be contacted for this purpose.

## Ethics Statement

This trial was approved by the Medical Ethics Committee of the Erasmus Medical Center in Rotterdam, the Netherlands (06-045). The patients/participants provided their written informed consent to participate in this study.

## Author Contributions

MW and WH contributed to the study design (patient selection, endpoint definitions, dose mapping procedures, statistical analyses) and writing of the drafts of the manuscript. LI and FP contributed to patient inclusion and follow-up. MW and BH contributed to data collection of dosimetric data. All authors contributed to critical reading, revision of the manuscript, and approval of the submitted version.

### Conflict of Interest

The authors declare that the research was conducted in the absence of any commercial or financial relationships that could be construed as a potential conflict of interest.
