# Supplementary material for: Local Dose Effects for Late Gastrointestinal Toxicity After Hypofractionated and Conventionally Fractionated Modern Radiotherapy for Prostate Cancer in the HYPRO Trial
Source: Front Oncol. 2020 Apr 3;10:469. doi: 10.3389/fonc.2020.00469 (PMC7169424; doi:10.3389/fonc.2020.00469)

Fig 1a. Average dose surface histograms with its standard deviation for “Total rectum” per treatment group, for the total treatment: physical dose (α/β=infinite*). Abbreviations*: HF=hypofractionation, SF=standard fractionation**.**

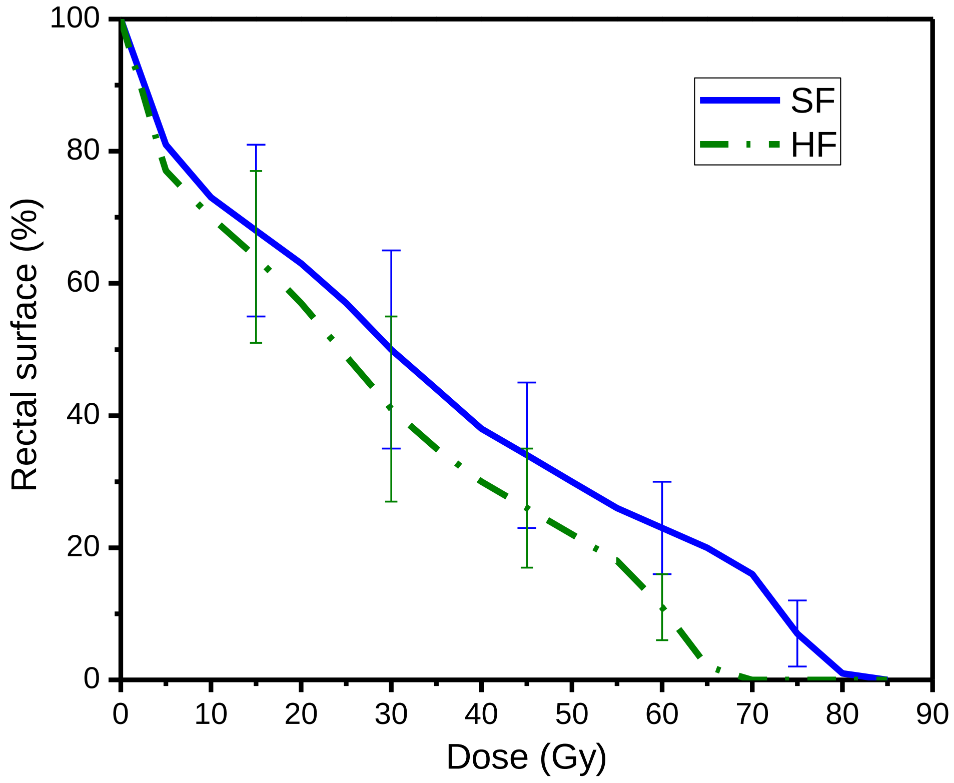

Fig 1b. Average dose surface histograms with its standard deviation for “Total rectum” per treatment group, for the total treatment with α/β=3*. Abbreviations*: HF=hypofractionation, SF=standard fractionation**.**

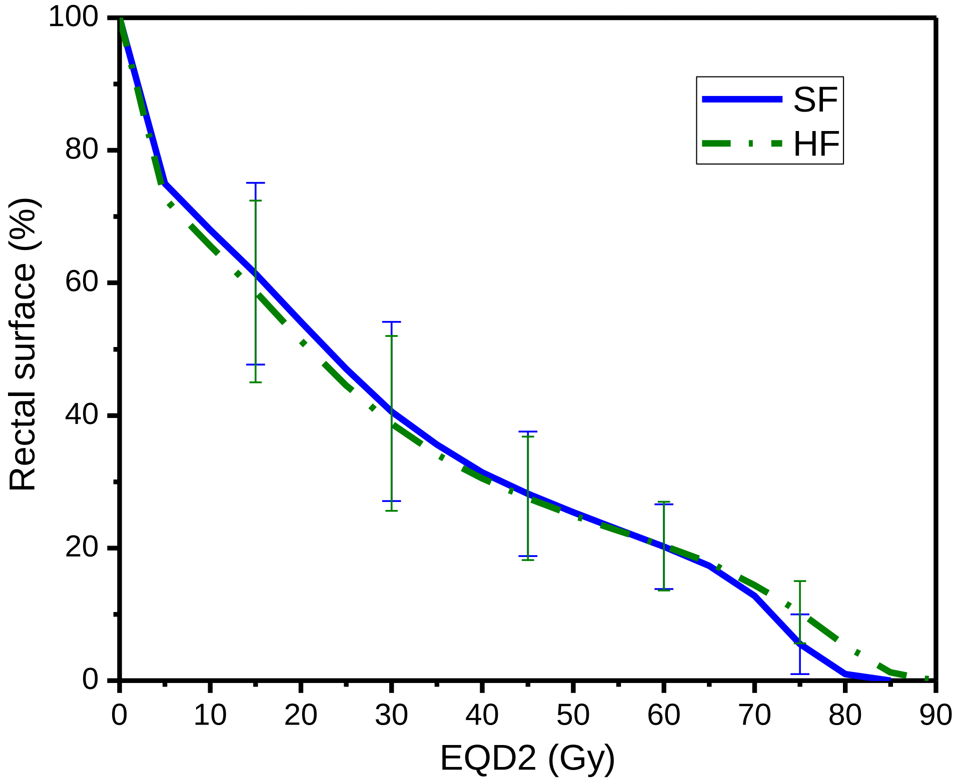

Supplement: Supplementary file 1 [file Data_Sheet_1.PDF]
